# Endotoxaemia in Obstructive Jaundice

**DOI:** 10.1155/1991/48672

**Published:** 1991

**Authors:** Thomas Diamond, Brian J. Rowlands

**Affiliations:** 1 Department of Surgery, The Queen's University of Belfast Ireland; 2 Department of Surgery Institute of Clinical Science Grosvenor Road Belfast BT12 6BJ Ireland


*HPB Surgery*, 1991, Vol. 4, pp. 81–94
Reprints available directly from the publisher
Photocopying permitted by license only

© 1991 Harwood Academic Publishers GmbH
Printed in the United Kingdom

### REVIEW ARTICLE

### ENDOTOXAEMIA IN OBSTRUCTIVE JAUNDICE

THOMAS DIAMOND and BRIAN J.ROWLANDS

*Department of Surgery, The Queen's University of Belfast*

*(Received 17 December 1990)*

KEY WORDS: Bile duct obstruction, extrahepatic, endotoxin, Limulus test, bile salts, endotoxin antibodies, biliary drainage

### INTRODUCTION

Surgical procedures in patients with obstructive jaundice are associated with significant morbidity and mortality[1]. This is due, to a large extent, to the development of postoperative complications such as sepsis, bleeding disorders and renal failure[2-4]. Clinical and experimental studies have suggested several aetiological factors for these complications including hypotension, impaired nutritional status, impaired immune function and the presence of potential toxic substances in the circulation such as bilirubin and bile acids[1,5,6]. However, in recent years, there has been an increasing recognition of the role of circulating endotoxins in the development of complications in obstructive jaundice[4,7-9].

The focus of this review will be the proposed association between the presence of systemic endotoxaemia in obstructive jaundice and the subsequent development of systemic complications. The experimental and clinical evidence for the existence of portal and systemic endotoxaemia in obstructive jaundice will be reviewed, followed by an outline of the current theory on the origin and mechanisms of development of endotoxaemia. The effects of endotoxaemia in jaundiced animals and patients, and the mechanisms by which these may be produced, will then be reviewed. The recognition of endotoxaemia as a possible cause of complications in obstructive jaundice and other pathological situations has led to the development of a variety of therapeutic strategies, including the use of anti-endotoxin agents, and these will be discussed.

### *Is the Development of Endotoxaemia in Obstructive Jaundice a Definite Pathological Entity?*

Since the association between the presence of endotoxaemia and renal failure in

Address correspondence to: Professor B J Rowlands, Department of Surgery, Institute of Clinical Science, Grosvenor Road, Belfast BT12 6BJ

obstructive jaundice was reported by Wardle and Wright in 1970, several animal and clinical studies have been performed to detect endotoxin in the circulation in obstructive jaundice[7]. The significance of the results of these studies obviously depends on the accuracy and reproducibility of the method used for detection of endotoxin.

In 1956, Bang reported that a lysate derived from blood cells (amoebocytes) of the horseshoe crab (Limulus Polyphemus) clotted in the presence of minute amounts of gram-negative bacterial endotoxin[10]. This Limulus amoebocyte lysate (LAL) reaction has formed the basis for the Limulus assay used to detect serum endotoxin. The LAL reaction involves the activation, by endotoxin, of a proenzyme in the Limulus lysate. This results in release of a clotting enzyme which reacts with a protein, derived from sheep erythrocytes, resulting in the formation of a gel. This is the basis of the standard Limulus assay which, therefore, provides a qualitative detection of the presence of serum endotoxin[11].

In 1978, Iwanga *et al.* reported that the activated LAL proenzyme (clotting enzyme referred to in the standard assay) would cleave p-nitroaniline (pNA) substrates and that this could be used as a quantitative assay for endotoxaemia[12]. The principle of the quantitative assay is that the enzyme formed from the proenzyme in the LAL, following activation by endotoxin, catalyses the splitting of pNA from a chromogenic substrate. The release of pNA is directly proportional to endotoxin concentration and is measured photometrically. The amount of enzyme needed to split the chromogenic substrate is much less than the amount needed to form a clot, as in the standard assay. The chromogenic assay is, therefore, approximately 100 times more sensitive than the standard assay and is capable of detecting endotoxin in concentrations as low as 10 pg/ml[13].

There are several problems with the Limulus reaction which have not been solved by the development of the quantitative assay. These are related principally to the presence of proteins in plasma (e.g. esterases, elastases, anti-thrombin III) which influence and inhibit the reaction between endotoxin and the LAL[13-15]. Several methods have been employed to inactivate or remove these but the preferred technique is dilution and heat treatment[13,15-18]. In addition, endotoxin may be denatured rapidly if blood is not kept at 4°C immediately after sampling and problems with the assay often occur as a result of exogenous contamination with endotoxin[18,19]. This, in addition to the natural variability of the LAL, means that there is often a high batch variability and poor reproducibility of the assay[18,19].

For these reasons, results of studies utilising the Limulus assay may be variable and must always be interpreted with great care. The results of animal and clinical studies reported to date are summarised in Table 1. The vast majority of studies report a positive correlation between the presence of obstructive jaundice and the development of significant portal and/or systemic endotoxaemia, but in two of the animal studies, no significant portal or systemic endotoxaemia was detected. This is noteworthy as in these studies the chromogenic assay was used and if the development of endotoxaemia is a definite pathological entity, it is surprising that neither significant portal nor systemic endotoxaemia was detected[30,31]. However, despite these two contradictory reports, the majority of experimental data supports the development of endotoxaemia and in many of the studies, a definite relationship between endotoxaemia and complications was also demonstrated.

**Table 1** Summary of studies investigating the development of endotoxaemia in obstructive jaundice.

**Significant portal and/or systemic endotoxaemia detected**

**Animal studies**
Bailey 1976[8].
Gouma *et al.* 1986[20].
Diamond *et al.* 1990[21].

**Human studies**
Wilkinson *et al.* 1974[22].
Wardle 1974[23].
Wilkinson *et al.* 1976[4].
Bailey 1976[8].
Hunt *et al.* 1982[3].
Cahill 1983[9].
Ingoldby 1984[26].
Pain and Bailey 1986[27].
Gawley *et al.* 1988[29].

**Significant portal and systemic endotoxaemia NOT detected**

**Animal studies**
Roughneen *et al.* 1988[30].
Greve *et al.* 1990[31].

### *Origin and Mechanism of Development of Endotoxaemia*

The largest reservoir of gram-negative organisms in the body is the large bowel[32]. This is, therefore, the largest potential site from which endotoxaemia may originate but, theoretically, any infected focus or duct system in the body could act as a source of endotoxaemia. While the large bowel is thought to be the most likely source of endotoxaemia in obstructive jaundice, it should be remembered that a significant proportion of jaundiced patients have infection in the biliary system particularly if an invasive procedure has been performed. This may be another potential source of endotoxin[33].

It is postulated that, in patients with obstructive jaundice, there are two major contributing factors in the development of endotoxaemia. Firstly, the absence of bile salts in the gut results in an alteration of the bacterial flora and loss of the emulsifying anti-endotoxin effect of bile salts results in a larger pool of endotoxin in the large bowel, for absorption into the portal circulation[8,34,35]. Secondly, impairment of hepatic Kupffer cell phagocytic function in obstructive jaundice allows spillover of endotoxin into the systemic circulation with subsequent development of systemic complications (Figure 1)[36-38,39].

This hypothesis provides a simple model for the understanding of the development of endotoxaemia in biliary obstruction, but the situation is likely to be more complex. Other factors affecting enteric endotoxin production and portal absorption may be important. Anti-endotoxin secretory IgA produced from the liver, which contributes significantly to gut immunity, is reduced in obstructive jaundice[40], and other non-specific factors such as changes in mucosal permeability, effects of hyperbilirubinaemia and changes in mucosal blood flow may all affect the integrity of the bowel wall as a mechanical and physiological barrier to the passage of bacteria and toxins into the portal circulation[41,42].

This phenomenon of passage of bacteria or bacterial toxins from the gut lumen is known as bacterial translocation and has been shown to occur experimentally under a variety of circumstances such as mucosal damage, bowel ischaemia, trauma,

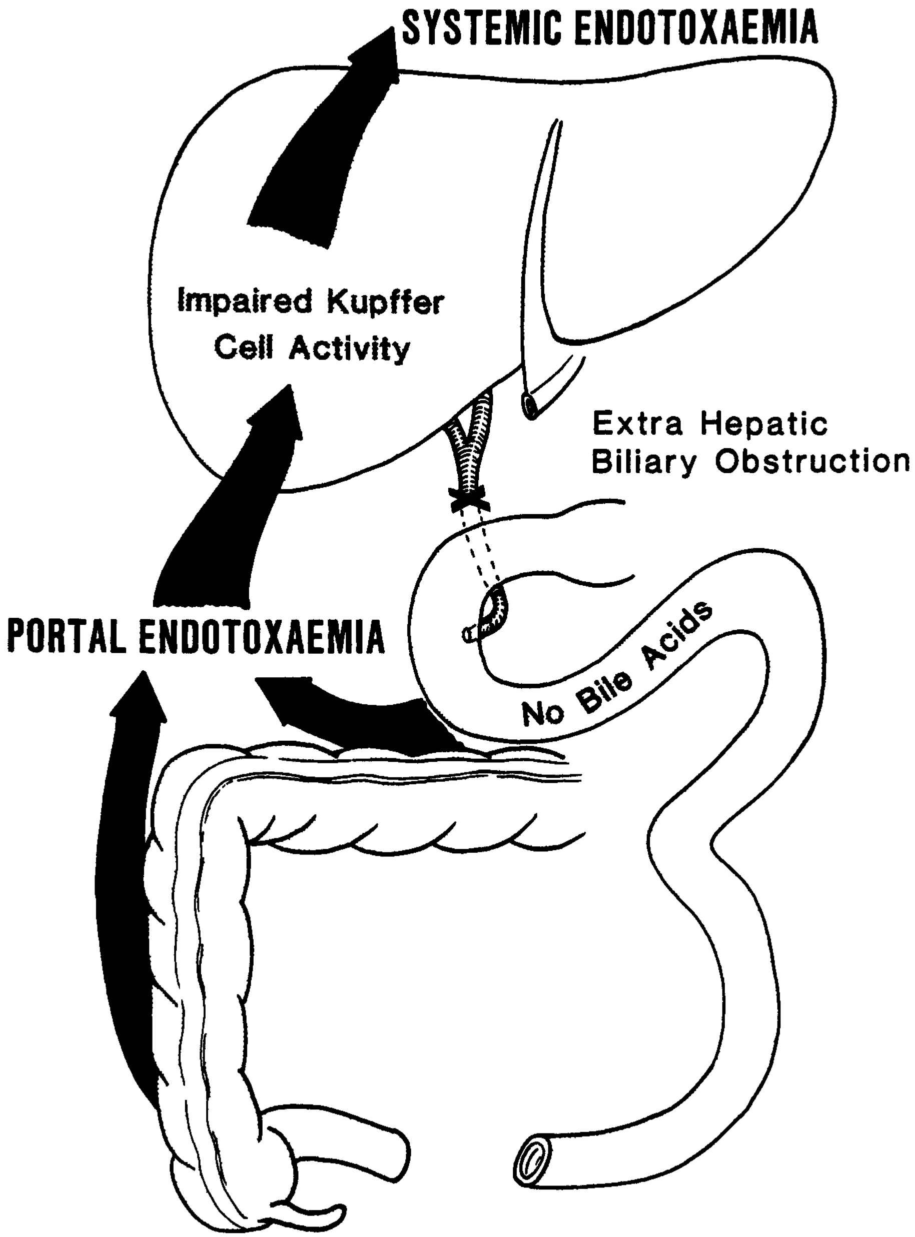

**Figure 1** Proposed origin and mechanism of development of endotoxaemia in obstructive jaundice.

burns, systemic administration of endotoxin and obstructive jaundice[43-45]. It seems from some of these studies that circulating endotoxin itself is one of the factors which can alter gut permeability and in obstructive jaundice, lack of bile flow and other factors predispose to portal absorption of endotoxin and impaired reticuloendothelial function allows systemic endotoxaemia to develop. This further impairs the gut barrier to portal absorption of endotoxin and a vicious positive feedback circle is entered which ultimately leads to the development of overwhelming systemic endotoxaemia.

### *Effects of Endotoxaemia in Obstructive Jaundice*

### *Renal failure*

Approximately 65% of patients with obstructive jaundice develop impaired renal function following surgery and in 10–20% renal failure occurs[6,8,9,46,47]. Proposed aetiological factors, based on animal and human experiments include hypovolaemia, hypotension and possible toxic agents such as bilirubin and bile salts but, in recent years, studies have concentrated on the role of endotoxaemia as a causative factor[6-9]. There is a strong correlation in human and animal studies between the presence of endotoxins in the systemic circulation and renal impairment[4,7-9,26]. Furthermore, renal impairment is rare in the absence of endotoxaemia[4,8].

Endotoxin causes a marked increase in renal vascular resistance, possibly as a result of renal fibrin deposition due to intravascular coagulation[47]. Affected kidneys show glomerular and peritubular fibrin deposition, leading to acute tubular necrosis. These changes are similar to those seen in the Schwartzmann reaction produced by repeated injections of endotoxin in animals and this is a proposed mechanism of acute renal failure in obstructive jaundice[7].

### *Sepsis*

Infective complications such as wound infection, septicaemia and peritonitis occur more frequently in jaundiced patients and contribute largely to the reported high morbidity and mortality[2,5,48,49]. Impairment of immune function has been suggested as a possible cause. This is based on the demonstration in several animal and human studies of impairment of both reticuloendothelial function and cellular immune function[37,50]. Thus, endotoxaemia may have a causative role in the development of infective complications as endotoxin is known to alter immune function[32,51].

### *Wound healing*

Impaired wound healing, as measured by bursting strength, has been demonstrated in animals with obstructive jaundice[53,54]. In jaundiced patients, incisional herniae have been shown to occur in approximately 10% and wound dehiscence in approximately 3% of patients[48,52].

The exact cause of this apparent impairment of wound healing in obstructive jaundice has not been fully elucidated. The presence of malignancy and poor nutritional status are thought to be significant factors[52,55]. It has also been suggested that impaired wound healing may be associated with endotoxaemia as improved wound healing has been demonstrated in jaundiced rats treated with oral bile salts, a therapy said to prevent endotoxaemia[1,56].

*Coagulation disorders*

Postoperative haemorrhage is a significant complication following surgery in jaundiced patients[5,49,76]. This was initially thought to be due to vitamin K deficiency as a result of malabsorption of the fat soluble vitamins[57]. However, despite normal coagulation, some patients develop postoperative gastrointestinal haemorrhage due to acute gastric erosions[58]. Animal studies demonstrate that endotoxin administration induces similar gastric lesions and it has been suggested that these are caused by endotoxaemia in patients[59,60].

Disseminated intravascular coagulation (DIC)has been reported in obstructive jaundice, particularly in association with biliary tract infection and endotoxaemia[3,61]. Wardle reported decreased fibrinolytic activity in jaundiced patients but Hunt *et al.* reported the presence, in serum, of increased fibrinogen degradation products (FDP), associated with endotoxaemia and post operative mortality[3,23].

There are, therefore, several suggested explanations for the haemorrhagic complications seen in jaundiced patients. Firstly, prolongation of the prothrombin time caused by malabsorption of vitamin K and impaired ability of the liver to manufacture prothrombin. Secondly, an increased fibrinolytic activity leading to ineffective coagulation and thirdly, the presence of gastric erosions. The latter two phenomena are thought to be due to endotoxaemia.

*Multiple system organ failure*

The progressive deterioration of function in organ systems, either simultaneously or sequentially, is known as organ failure[62-64]. The syndrome of multiple system organ failure (MSOF) occurs following failure of two or more major organ support systems[62]. Failure of the respiratory, renal and cardiovascular systems is the most frequently encountered combination but failure of other vital support functions including the gastrointestinal. hepatic, coagulation and central nervous systems are important components of the MSOF syndrome. Sepsis is the principal activating factor in the development of MSOF but it is also thought that endotoxaemia is an important aetiological factor[63,64].

When the features of the multiple system organ failure syndrome are compared to the complications in obstructive jaundice, there is a very strong similarity between the two clinical situations. It seems possible, therefore, that organ failure in obstructive jaundice and the multiple system organ failure syndrome are similar entities. Not only are the two clinical situations relatively similar but similarities in the origin of these also exist i.e. the presence of sepsis and endotoxaemia.

### *Mechanism of Endotoxin-induced Complications*

How does the presence of systemic endotoxaemia produce failure of individual organs and organ systems? The effects of endotoxaemia on the host are so widespread that it seems unlikely that they could all be produced by endotoxin directly. It is thought that endotoxin acts as the afferent side or activating agent of an inflammatory response which leads ultimately to impairment of organ function. Other activating agents of this inflammatory cascade are bacterial infection and severe tissue injury with necrosis. This concept of activation, mediation and end organ effects has been popularised by Cerra to help understand how an insult such as sepsis, trauma or endotoxaemia can produce such widespread effects as the multiple system organ failure syndrome and is also a very useful model for explaining the consequences of endotoxaemia in obstructive jaundice[65].

*Biological mediators*
Activation of the inflammatory response leads to the release of a vast array of inflammatory mediators. These may be released from endothelial cells but the majority are released from leucocytes such as neutrophils and particularly from cells of the monocyte-macrophage system. All of these mediators are part of normal host defence but may become overstimulated and produce organ dysfunction through complex interactions between cells and mediators. Examples include oxygen radicals, serotonin, histamine, fibronectin and more recently discovered cytokines such as leucotrienes, interleukins, eicosanoids and tumour necrosis factor (TNF)[66,67].

The eicosanoids are a group of products which are formed from arachidonic acid. Metabolism via the cyclooxygenase pathway produces the prostaglandins and these are thought to be important as mediators of the effects of endotoxin in obstructive jaundice. Significant elevation of plasma thromboxane and inhibition of 6-oxo-prostaglandin F1-alpha has been demonstrated following endotoxin administration and bile duct ligation in rats[47,68].

*End organ effects*
The mechanisms by which the biological mediators engage the efferent or effector side of the inflammatory cascade and produce impairment of organ function are referred to as end-organ effects. These include changes in the microcirculation, cell membrane permeability, oxygen delivery, cellular metabolism, vascular permeability and activation of the coagulation system with resultant microembolisation[51]. These changes occurring at a cellular and microvascular level ultimately produce alterations in organ function such as impaired glomerular filtration in the kidney leading to renal failure or impaired oxygen transport in the lungs leading to respiratory failure and the adult respiratory distress syndrome[4,5,7,66].

### ANTI-ENDOTOXIN THERAPY IN OBSTRUCTIVE JAUNDICE

*Biliary Drainage*

Preoperative, percutaneous external biliary decompression was introduced to prepare patients for definitive surgery for obstructive jaundice but prospective clinical trials failed to show any significant improvement in morbidity or mortality following the use of this technique[69-72]. A study in rats demonstrated that following relief of biliary obstruction by internal drainage, endotoxaemia was reduced but following external drainage, endotoxaemia was unaffected[20]. Thus, it was proposed that failure of external drainage in jaundiced patients was due to persistence of endotoxaemia, as a result of failure of return of bile to the gastrointestinal tract[20].

However, using a technique for sterile, uncomplicated external biliary drainage in the rat, it has been demonstrated that endotoxaemia was reversed by both internal and external biliary drainage[21,73]. It has, therefore, been suggested that return of bile to the gut may not be as important in the prevention of endotoxaemia, as previously supposed, and failure of external drainage in animal and patient studies may have been due to local complications associated with the drainage cannula, such as bleeding, bile leakage and the development of local and systemic infection[21]. The importance of these complications has also been highlighted in several clinical studies[33,71,72].

In patients, it should be possible to avoid some of the complications associated with percutaneous liver puncture and drainage by performing endoscopic internal biliary drainage. Improvement in complication rates and mortality following pancreatic resection have been demonstrated using this technique[28]. However, no studies on the efficacy of endoscopic biliary decompression in reversing endotoxaemia have been reported.

*Large bowel irrigation*

As the large bowel is thought to be the site of origin of endotoxin in obstructive jaundice, it has been suggested that irrigation and lavage to reduce bacterial numbers could be beneficial[74]. This has been shown to be beneficial in prevention of endotoxaemia in patients with inflammatory bowel disease but in jaundiced patients studied by Hunt, bowel preparation did not produce any significant benefit[75,76].

*Bile salts*

Bile salts have antibacterial effects and a direct detergent effect on the lipopolysaccharide endotoxin molecule and are thought to play an important role in the defence against bacterial endotoxins[77,78]. The theoretical basis for their oral administration in obstructive jaundice is that they replace the normal protective anti-endotoxin effect of bile which is lost in complete biliary obstruction and, therefore, help reduce the endotoxin pool in the large bowel.

The potential therapeutic effect of oral bile salts may be due to a direct anti-endotoxin effect as it has been reported that biliary diversion does not alter gastrointestinal flora and that there is no alteration in the gut flora of jaundiced patients[9,78,79]. However, in a recent study in mice, in which caecal bacterial populations were quantified, the levels of gram-negative enteric bacilli were 100-fold higher in bile duct ligated animals compared to sham operated and control groups[44]. The potential therapeutic effect of bile salts may, therefore, be due to a combination of an anti-bacterial effect and a direct anti-endotoxin effect.

Koscar *et al.* showed that endotoxin absorption was increased in rats in the absence of bile salts and oral administration of bile salts has shown to significantly reduce mortality following endotoxin administration in jaundiced rats[3,34].

In patients, preoperative oral bile salt administration has been reported to prevent systemic endotoxaemia and protect postoperative renal function[9,79]. However, a prospective, randomised clinical trial of oral ursodeoxycholic acid in obstructive jaundice showed no significant benefit in terms of systemic endotoxaemia, renal function or postoperative outcome[80]. In the study by Cahill deoxycholic acid was used and in the study by Evans *et al.* taurocholate was used[9,79]. Gawley *et al.* reported that sodium deoxycholate reduced endotoxaemia in jaundiced patients but in contrast to the previous study by Cahill an impaired renal function was actually found in the bile salt treated patients[9,29].

Recent studies have shown that ursodeoxycholic acid is much less potent as an anti-endotoxin agent than deoxycholic acid and this was proposed as an explanation for the poor results of the clinical trial carried out by Thompson *et al.*[9,80,81].

However, this does not explain the impaired renal function observed in the group of patients treated with sodium deoxycholate by Gawley *et al*[29].

The results of studies of oral bile salts, endotoxaemia and renal function in patients with obstructive jaundice are conflicting and no definitive conclusion on the efficacy of their use can be made. One important aspect which is not addressed in any of these papers is the fact that, in the gastrointestinal tract, the vast majority of intraluminal bile salts are absorbed in the terminal ileum (enterohepatic circulation). For example, less than 5% of deoxycholate actually enters the colon[82]. If the colon acts as a reservoir of bacteria and endotoxins, it seems unlikely that such a small percentage of bile salts entering the colon could have an important protective effect in the normal individual. In addition, in the patient with obstructive jaundice, administered bile salts may be absorbed in the terminal ileum and not reach the colon — the site which is proposed to be the major source of endotoxin[1,8]. This factor, in addition to the conflicting results of the studies mentioned above, would seem to be the major criticism of the theoretical basis for advocating preoperative bile salt administration in patients with obstructive jaundice.

*Lactulose*

Oral lactulose has been shown to have anti-endotoxin effects in animals and is used in the treatment of patients with endotoxaemia secondary to liver disease[24,83-85].

Lactulose reduces endotoxin related mortality and improves survival in bile duct ligated rats[27,87]. It has also been reported to reduce endotoxaemia and protect renal function in jaundiced patients and preoperative oral lactulose therapy is now recommended in patients with obstructive jaundice[27].

*Prostaglandin manipulation*

Pharmacological manipulation of the thromboxane/prostacyclin ratio by pretreatment with indomethacin, dazoxiben and prostacyclin improves renal fibrin deposition and survival and prevents the increase in urinary prostaglandin excretion usually seen following bile duct ligation[47,68]. However, no follow-up study has been performed in patients.

*Polymyxin*

The cationic polypeptide antibiotic polymyxin B has anti-endotoxin properties[88]. Ingoldby demonstrated improved survival following an endotoxin challenge in jaundiced rats treated with polymyxin B but a follow-up study in jaundiced patients failed to demonstrate any beneficial effect[26,89].

*Taurolidine*

Taurolidine is an antimicrobial compound with an anti-endotoxin effect. It acts by a chemical reaction, releasing methylol groups ($CH_2OH$) which become available to bind to and inactivate bacteria or endotoxin as taurolidine is metabolised through tauroltam to taurinamide and eventually to taurine[90]. Taurolidine has been shown to improve survival following endotoxin administration in jaundiced rats and to reduce endotoxaemia in experimental biliary obstruction but, to date, there are no reports of clinical trials of its efficacy in patients with obstructive jaundice[91,92].

*Endotoxin antibodies*

The continuing high mortality from gram-negative septicaemia and shock, despite the introduction of new and powerful antibiotics, has stimulated interest in the concept of anti-endotoxin immunotherapy. Two different approaches have been used to obtain antiserum with a wide range of anti-endotoxin activity. Antibodies have been developed against the oligosaccharide side chain of endotoxin (anti-LPS)[25]. The problem with these is that the 0 side chain is "strain specific" and antibody developed against endotoxin from one strain of bacteria would not be effective against that from another.

Because the toxic effects of endotoxin are associated with the core glycolipid region, in which there is little strain variation, development of antibodies against the core glycolipid region protects against a wide variety of gram-negative bacteria and endotoxins[93,94]. These antibodies are raised using so called "rough" mutant strains of gram-negative bacteria (e.g. Escherichia Coli J5 and Salmonella Minnesota R595). This may represent a possible therapeutic strategy in patients with obstructive jaundice but to date, no studies of the efficacy of anti-endotoxin immunotherapy in preventing complications associated with endotoxaemia in obstructive jaundice have been reported.

*Antibiotics*

Oral, non-absorbable antibiotics, such as neomycin, may decrease the colonic bacterial population but this may be detrimental in that bacterial death may actually increase the amount of free endotoxin available for portal absorption[86]. Preoperative oral antibiotics are, therefore, not recommended as an anti-endotoxin therapy in obstructive jaundice[1].

### CONCLUSIONS

This review has focused on the proposed development of endotoxaemia in obstructive jaundice. The problems with the assay used to detect endotoxin and the necessity for cautious interpretation of results is emphasised. Despite these reservations, the majority of animal and clinical studies, reported to date, support the theory of development of significant portal and systemic endotoxaemia and subsequent systemic complications in obstructive jaundice. Several therapeutic strategies have been proposed but, so far, no single anti-endotoxin therapy has emerged as a very significant breakthrough in the management of obstructive jaundice. Further study of these, including prospective clinical trials employing single or multiple strategies for the prevention of endotoxaemia, are required.

An additional requirement to further elucidate the mechanisms of the complications attributed to endotoxaemia, is a more specific and reproducible assay for the detection of endotoxin. Modification of this assay to allow detection of endotoxin in other body fluids, such as bile, would allow assessment of the significance of infection in the biliary tree as a potential souce of endotoxaemia. Further investigation of other possible therapeutic measures in obstructive jaundice, such as biliary drainage, enhancement of immune function and pharmacological manipulation of inflammatory mediators, is also indicated.

*References*

1. Pain, J.A., Cahill, C.J. and Bailey, M.E. (1985) Perioperative complications in obstructive jaundice: therapeutic considerations. *Br. J.Surg.*, **72**, 942–945

2. Armstrong, C.P., Dixon, J.M., Taylor, T.V. and Davies, G.C. (1984) Surgical experience of deeply jaundiced patients with bile duct obstruction. *Br.J.Surg.*, **71**, 234–238
3. Hunt, D.R., Allison, M.E.M., Prentice, C.R.M. and Blumgart, L.H. (1982) Endotoxaemia disturbance of coagulation and obstructive jaundice. *Am.J.Surg.*, **144**, 325–329
4. Wilkinson, S.P., Moodie, H., Stamatakis, J.D., Kakkar, V.V. and Williams R. (1976) Endotoxaemia and renal failure in cirrhosis and obstructive jaundice. *Br.Med.J.*, **2**, 1415–1418
5. Pitt, H.A., Cameron, J.L., Postier, R.G. and Gadacz, T.R. (1981) Factors affecting mortality in biliary tract surgery. *Am.J.Surg.*, **141**, 66–72
6. Wait, R.B. and Kahng, K.U. (1989) Renal failure complicating obstructive jaundice. *Am.J.Surg.*, **157**, 256–263
7. Wardle, E.N. and Wright, N.A. (1970) Endotoxin and acute renal failure associated with obstructive jaundice. *Br.Med.J.*, **4**, 472–474
8. Bailey, M.E. (1976) Endotoxin, bile salts and renal function in obstructive jaundice. *Br.J.Surg.*, **66**, 392–397
9. Cahill, C.J. (1983) Prevention of postoperative renal failure in patients with obstructive jaundice — the role of bile salts. *Br.J.Surg.*, **70**, 590–595
10. Bang, F.B. (1956) A bacterial disease of Limulus Polyphemus. *Bulletin of Johns Hopkins Hospital*, **98**, 325–350
11. Levin, J., Tomasulo, P.A. and Oser, R.S. (1970) Detection of endotoxin in human blood and demonstration of an inhibitor. *J.Clin.Lab.Med.*, **75**, 903–911
12. Iwanaga, S., Morita, T., Harada, T., Nakamura, S., Niwa, M., Takada, K., Kimura, T. and Sakakibara, S. (1978) Chromogenic substrates for horseshoe crab clotting enzyme. Its application for the assay of bacterial endotoxins. *Haemostasis*, **7**, 183–188
13. Cohen, J., McConnell, J.S. (1984) Observations on the measurement and evaluation of endotoxaemia by a quantitative Limulus lysate microassay. *J.Infect.Dis.*, **150**, 916–924
14. Jacob, A.I., Goldberg, B.S. and Bloom, N. (1977) Endotoxin and bacteria in portal blood. *Gastroenterol.*, **72**, 1268–1270
15. Scarnes, R.C. (1966) The inactivation of endotoxin after interaction with certain proteins of normal serum. *Ann.N.Y.Acad.Sci.*, **133**, 644–662
16. Fink, P.C., Lehr, L. and Urbaschek, R.M. (1981) Limulus amoebocyte lysate test for endotoxaemia: investigations with a sensitive spectrophotometric assay. *Klin.Wochenschr.*, **59**, 213–218
17. Sturk, A., Van Deventer, S.J.H., ten Cate J.W. and Buller, H.R. (1988) In: *Vandeventer S J H*, MD Thesis, University of Amsterdam, 53–73
18. Thomas, L.L.M., Sturk, A., Kahle, L.H. and ten Cate, J.W. (1981) Quantitative endotoxin determination in blood with a chromogenic substrate. *Clin.Chim.Acta.*, **116**, 63–68
19. Diamond, T. (1991) Experimental aspects of endotoxaemia in obstructive jaundice: MD Thesis. The Queen's University of Belfast
20. Gouma, D.J., Coelho, J.C.U., Fisher, J.D., Schelegel, J.F., Li, Y.F. and Moody, F.G. (1986) Endotoxaemia after relief of biliary obstruction by internal and external drainage in rats. *Am.J.Surg.*, **151**, 476–479
21. Diamond, T., Dolan, S., Thompson, R.L.E. and Rowlands, B.J. (1990) Development and reversal of endotoxaemia and endotoxin — related death in obstructive jaundice. *Surgery*, **108**, 370–375
22. Wilkinson, S.P., Arroyo, V., Gazzard, B.G., Moodie, H. and Williams, R. (1974) Relation to renal impairment and haemorrhagic diathesis to endotoxaemia in fulminant hepatic failure. *Lancet*, **i**, 521–524
23. Wardle, E.N. (1974) Fibrinogen in liver disease. *Arch.Surg.*, **109**, 741–746
24. Iwasaki, M., Maruyama, I., Ikeziri, N. Tanikawa, K. (1980) Endotoxin in severe liver disease. *Jap.J.Gastroenterol.*, **77**, 386–394
25. Baumgartner, J.D. and Glauser, M.P. (1987) Controversies in the use of passive immunotherapy for bacterial infections in the critically ill patient. *Rev.Infect.Dis.*, **9**, 194–205
26. Ingoldby, C.J., McPherson, G.A.D. and Blumgart, L.H. (1984) Endotoxaemia in obstructive jaundice. Effect of polymyxin B. *Am.J.Surg.*, **147**, 766–771
27. Pain, J.A. and Bailey, M.E. (1986) Experimental and clinical study of lactulose in obstructive jaundice. *Br.J.Surg.*, **73**, 775–778
28. Trede, M. and Schwall, G. (1988) The complications of pancreatectomy. *Ann.Surg.*, **207**, 39–41
29. Gawley, W.F., Gorey, T.F., Johnson, A.H., Collins, P.B., Osborne, D.H., Lane, B.E. and Collins, P.G. (1988) The effect of oral bile salts on serum endotoxin and renal function in obstructive jaundice. *Br.J.Surg.*, **75**, 600

30. Roughneen, P.T., Kumar, S.C., Pellis, N.R. and Rowlands, B.J. (1988) Endotoxaemia and cholestasis. *Surg.Gynae.Obstet.*, **167**, 205–210
31. Greve, J.W., Gouma, D.J., Soeters, P.B. and Burmann, W.A. (1990) Suppression of cellular immunity in obstructive jaundice is caused by endotoxins. A study with germfree rats. *Gastroenterol.*, **98**, 478–485
32. Nolan, J.P. (1981) Endotoxin, reticuloendothelial function and liver injury. *Hepatology*, **1**, 458–465
33. Blenkharn, J.L., McPherson, G.A.D. and Blumgart, L.H. (1984) Septic complications of percutaneous transhepatic biliary drainage. Evaluation of a new closed system. *Am.J.Surg.*, **147**, 318–321
34. Koscar, L.T., Bertok, L. and Varteresz, V. (1969) Effect of bile acids on intestinal absorption of endotoxin in rats. *J.Bacteriol.*, **100**, 220–223
35. Iwasaki, M. and Tanikawa, K. (1981) Liver diseases and endotoxin. Part 1. Effect of bile acid on endotoxin. *Nippon Shkakibyo Gakkai Zasshi*, **78**, 1232–1240
36. Drivas, G., James, O. and Wardle, N. (1976) Study of reticuloendothelial phagocytic capacity in patients with cholestasis. *Br.Med.J.*, **1**, 1568–1569
37. Holman, J.M. and Rikkers, L.F. (1982) Biliary obstruction and host defence failure.*J.Surg.Res.*, **32**, 208–213
38. Pain, J.A. (1987) Reticuloendothelial function in obstructive jaundice. *Br.J.Surg.*, **74**, 1091–1094
39. Bradfield, J.W.B. (1974) Control of spillover: the importance of Kupffer cell function in clinical medicine. *Lancet*, **ii**, 883–886
40. Rank, J.and Wilson, I.D. (1983) Changes in IgA following varying degrees of biliary obstruction in the rat. *Hepatology*, **3**, 241--247
41. Bennion, R.S., Wilson, S.E. and Williams R.A. (1984) Early portal anaerobic bacteraemia in mesenteric ischaemia. *Arch.Surg.*, **119**, 151–155
42. Papa, M., Halperin, Z., Rubenstein, E., Orenstein, A., Gafin, S. and Adar, R. (1983) The effect of ischaemia on the dog's colon on transmural migration of bacteria and endotoxin. *J.Surg.Res.*, **35**, 264–269
43. Deitch, E.A., Berg. R.D. and Specian, R. (1987) Endotoxin promotes the translocation of bacteria from the gut. *Arch.Surg.*, **112**, 185–190
44. Deitch, E.A., Sittig, K., Li, M. and Berg, R. (1990) Obstructive jaundice promotes bacterial translocation from the gut. *Am.J.Surg.*, **159**, 79–84
45. Berg, R.D. and Garlington, A.W. (1979) Translocation of certain indigenous bacteria from the gastrointestinal tract to the mesenteric lymph nodes and other organs in a gnotobiotic mouse model. *Infect.Immun.*, **23**, 403–411
46. Allison, M.E.M., Prentice, C.R.M., Kennedy, A.C. and Blumgart, L.H. Renal function and other factors in obstructive jaundice. *Br.J.Surg.*, **66**, 392–397
47. Fletcher, M.S., Westwick, J. and Kakkar, V.V. (1982) Endotoxin, prostaglandins and renal fibrin deposition in obstructive jaundice. *Br.J.Surg.*, **69**, 625–629
48. Blamey, S.L., Fearon, K.C.H., Gilmour, W.H., Osbourne, D.H. and Carter, D.C. (1983) Prediction of risk in biliary surgery. *Br.J.Surg.*, **70**, 535–538
49. Greig, J.D., Krukowski, Z.H. and Matheson, N.A. (1988) Surgical morbidity and mortality in one hundred and twenty nine patients with obstructive jaundice. *Br.J.Surg.*, **75**, 216–219
50. Roughneen, P.T., Gouma, D.J., Kulkarni, A.D., Fanslow, W.F. and Rowlands, B.J. (1986) Impaired specific cell-mediated immunity in experimental biliary obstruction and its reversibility by internal biliary drainage. *J.Surg.Res.*, **41**, 113–125
51. Morrison, D.C. and Ulevitch, R.J. (1978) The effects of bacterial endotoxins on host mediation systems. *Am.J.Pathol.*, **93**, 527–617
52. Armstrong, C.P., Dixon, J.M., Duffy, S.W., Elton, R.A. and Davies, G.C. (1984) Wound healing in obstructive jaundice. *Br.J.Surg.*, **71**, 267–270
53. Bayer, I. and Ellis, H. (1976) Jaundice and wound healing: an experimental study. *Br.J.Surg.*, **63**,
54. Arnaud, J.P., Humbert, W., Eloy, M.R. and Adloff, M. (1981) Effect of obstructive jaundice on wound healing. An experimental study in rats. *Am.J.Surg.*, **141**, 593–596
55. Irvin, T.T., Vassilakis, J.S., Chattopadhyay, D.K. and Greaney, M.G. (1978) Abdominal wound healing in jaundiced patients. *Br.J.Surg.*, **65**, 521–522
56. Askew, A.R., Bates, G.J. and Balderson, G. (1984) Jaundice and the effect of sodium taurocholate taken orally on abdominal wound healing. *Surg.Gynae.Obstet.*, **159**, 207–209
57. Wittenstein, B.H., Giacchino, J.L. and Pickleman, J.R. (1981) Obstructive jaundice: the necessity for improved management. *Am.Surg.*, **47**, 116–120

58. Dixon, J.M., Armstrong, C.P., Duffy, S.W., Elton, R.A. and Davies, G.C. (1984) Upper gastrointestinal bleeding. A significant complication after surgery for relief of obstructive jaundice. *Ann.Surg.*, **199**, 271–275
59. Griesman, S.E. and Woodward, C.L. (1970) In vivo studies on the role of liver in endotoxin fever and tolerance. *J.Clin.Invest.*, **49**, A37
60. Clements, C., Bosch, J., Rodes, J., Arroyo, V., Mas, A. and Margall S. (1977) Functional renal failure and haemorrhagic gastritis associated with endotoxaemia and cirrhosis. *Gut*, **18**, 556–560
61. Kunz, F., Amor, H., Hortnagl, G., Weiser, F., Foltznecht, K. Braunsteiner, H. (1974) Disseminerte intravaskulare Gerinnung und letale Makrotheombosierung bei einem Patienten mit Gallenwegskarzinom. *Dtsch.Med.Wschr.*, **99**, 2643–2647
62. Henao, F.and Alderete, J.S. (1985) Multiple systems organ failure: Is it a specific entity? *South Med.J.*, **78**, 329–334
63. Carrico, C.J., Meakins, J.L., Marshall, J.C., Fry, D. and Maier, R.V. (1986) Multiple organ failure syndrome. *Arch.Surg.*, **121**, 196–203
64. Fry, D.E., Garrions, R.N., Heitsch, R.C., Calhoun, K. and Polk, H.C. (1980) Determinants of death in patients with intraabdominal abscess. *Surgery*, **88**, 517–523
65. Cerra, F.B. (1987) The hypermetabolism organ failure complex. *World J.Surg.*, **11**, 173–181
66. Dinarello, C.A. and Weir, J.W. (1987) Lymphokines. *N.Eng.J.Med.*, **317**, 940–945
67. Doran, J.E. and Lundsgaard-Hensen, P. (1988) Role of the reticuloendothelial system in the pathogenesis of organ failure. *Br.J.Hosp.Med.*, **39**, 221–225
68. O'Neill, P.A., Wait, R.B. and Kahng, K.U. (1990) Obstructive jaundice and renal failure in the rat: The role of renal prostaglandins and the renin-angiotensin system. *Surgery*, **108**, 356–362
69. Nakayama, T., Ikeda, A. and Okuda, K. (1978) Percutaneous transhepatic drainage of the biliary tract. Technique and results in 104 cases. *Gastroenterol.*, **74**, 554–559
70. Pitt, H.A., Gomes, A.S., Lois, J.F., Mann, L.L., Deutsch, L.S. and Longmire, W.P. (1985) Does preoperative percutaneous biliary drainage reduce operative risk or increase hospital cost? *Ann.Surg.*, **201**, 545–553
71. Hatfield, A.R.W., Terblanche, J., Fattar, S., Kernoff, L., Tobias, R., Girdwood, A.H., Harries-Jones, R. and Marks, I.N. (1982) Preoperative external biliary drainage in obstructive jaundice: A prospective controlled clinical trial. *Lancet*, **ii**, 896–899
72. McPherson, G.A.D., Benjhamin, I.S., Hodgson, H.J.F., Bowley, N.B., Allison, D.J. and Blumgart, L.H. (1984) Preoperative percutaneous transhepatic biliary drainage: the results of a controlled trial. *Br.J.Surg.*, **71**, 371–375
73. Diamond, T. and Rowlands, B.J. (1990) Choledochovesical fistula: a model for sterile external biliary drainage. *Surg.Res.Comm.*, **8**, 131–138
74. Blumgart, L.H. (1978) Biliary tract obstruction: new approaches to old problems. *Am.J.Surg.*, **135**, 19–31
75. Wellman, W., Fink, P.C. and Schmidt, F.W. (1984) Whole gut irrigation as antiendotoxinaemic therapy in inflammatory bowel disease. *Hepato-gastroenterol.*, **31**, 91–93
76. Hunt, D.R. (1980) The identification of risk factors and their application to the management of obstructive jaundice. *Aust.N.Z.J.Surg.*, **50**, 476–480
77. Taramina, D.F., Milner, K.C., Ribi, E. and Rudbach, J.H. (1968) Reactions of endotoxins and surfactants. Immunologic properties of endotoxins treated with sodium deoxycholate. *J.Immunol.*, **100**, 444–450
78. Shands, J.W. and Chun, P.W. (1980) The dispersion of gram-negative lipopolysaccharide by deoxycholate. *J.Biol.Chem.*, **225**, 1221–1226
79. Evans, H.R.J., Torreabla, V., Hudd, C. and Knight, M. (1982) The effect of preoperative bile salt administration on postoperative renal function in patients with obstructive jaundice. *Br.J.Surg.*, **69**, 706–708
80. Thompson, J.N., Cohen, J., Blenkharn, J.I., McConnell, J.S., Barr, J. and Blumgart, L.H. (1986) A randomised clinical trial of ursodeoxycholic acid in obstructive jaundice. *Br.J.Surg.*, **73**, 634–636
81. Pain, J.A. and Bailey, M.E. (1988) Prevention of endotoxaemia in obstructive jaundice — a comparative study of bile salts. *HPB Surgery*, **1**, 21–27
82. Hoffmann, A.F. (1983) The enterohepatic circulation of bile acids in health and disease. In: Sleisenger, M.H., Fordtran, J.S. Eds., Gastrointestinal disease: pathophysiology, diagnosis and management. W.B. Saunders: Philidelphia, 115–134
83. Liehr, H., English, G. and Rasenack, U. (1980) Lactulose — a drug with anti-endotoxin effect. *Hepato-gastroenterol*, **27**, 356–360
84. de Groot, G.H., Schlam, S.W., Batavier, P., Mass, H.C.M. and Schicht, I. (1983) Incidence of endotoxaemia in pigs with ischaemic hepatic necrosis treated by haemodialysis. *Hepato-gastroenterol*, **30**, 240–242
85. Scevola, D., Magliulo, E., Tripin, L., Benzi-Cipelli, R. and Bernaedi, R. (1979) Control of endotoxinemia in liver disease by lactulose and paromomycin. *Boll.1st Sieroter Milan*, **58**, 242–247
86. Goto, H. and Nakamura, S. Liberation of endotoxin from Escherichia coli by addition of antibiotics. *Japan J.Exp.Med.*, **50**, 35–43
87. Greve, J.W., Maessen, J.G., Tiebosch, T., Burmann, W.A. and Gouma, D.J. (1990) Prevention of postoperative complications in jaundiced rats. Internal biliary drainage versus oral lactulose. *Ann.Surg.*, **212**, 221–227
88. Hughes, A., Madan, B.R. and Parratt, J.R. (1981) Polymyxin B sulphate protects rats against the haemodynamic and metabolic effects of E. Coli endotoxin. *Br.J.Pharmac.*, **74**, 701–707
89. Ingoldby, C.J.H. (1980) The value of polymyxin B in endotoxaemia due to obstructive jaundice and mesenteric ischaemia. *Br.J.Surg.*, **67**, 565–567
90. Pfirrmann, R.W. and Leslie, G.B. (1979) The anti-endotoxin activity of taurolin in experimental animals. *J.Applied Bacteriol.*, **46**, 97–102
91. Carey, P.D., Feeley, T.M. Keane, F.B. and Tanner, W.A. (1988) Taurolidine reduces endotoxin-related mortality in the jaundiced rat. *Br.J.Surg.*, **75**, 1253–1254
92. Diamond, T., Blair, P.H.B., McGlone, E. and Rowlands, B.J. Taurolidine reduces endotoxaemia in experimental biliary obstruction. 33rd World Congress of Surgery, *Abstracts*, 53
93. Ziegler, E.J., McCutchan, A.E., Fierer, J., Glauser, M.P., Sadoff, J.C., Douglas, H. and Braude, A.I. (1982) Treatment of gram-negative bacteraemia and shock with human antiserum to a mutant Escherichia coli. *N.Engl.J.Med.*, **307**, 1225–1230
94. Baumgartner, J.D., Glauser, M.P., McCutchan, J.A., Ziegler, E.J., Melle, G., Klauber, M.R., Vogt, M., Mulchen, E., Luethy, R., Chiolero, R. and Geroulanos, S. (1985) Prevention of gram-negative shock and death in surgical patients by antibody to endotoxin core glycolipid. *Lancet*, **ii**, 59–63

